# A New Landscape of Multiple Dispersion Kinks in a High-*T*_c_ Cuprate Superconductor

**DOI:** 10.1038/s41598-017-04983-0

**Published:** 2017-07-06

**Authors:** H. Anzai, M. Arita, H. Namatame, M. Taniguchi, M. Ishikado, K. Fujita, S. Ishida, S. Uchida, A. Ino

**Affiliations:** 1 Graduate School of Engineering, Osaka Prefecture University, Sakai, 599-8531 Japan; 2 Hiroshima Synchrotron Radiation Center, Hiroshima University, Higashi-Hiroshima, 739-0046 Japan; 3 Graduate School of Science, Hiroshima University, Higashi-Hiroshima, 739-8526 Japan; 4 Research Center for Neutron Science and Technology, Comprehensive Research Organization for Science and Society (CROSS), Tokai, Naka, Ibaraki 319-1106 Japan; 5 Laboratory for Atomic and Solid State Physics, Department of Physics, Cornell University, Ithaca, New York 14853 USA; 6 Advanced Industrial Science and Technology, Tsukuba, Ibaraki 305-8568 Japan; 7 Department of Physics, University of Tokyo, Tokyo, 113-0033 Japan; 8 Condensed Matter Physics and Materials Science Department, Brookhaven National Laboratory, Upton, NY 11973 USA

## Abstract

Conventional superconductivity is caused by electron-phonon coupling. The discovery of high-temperature superconductors raised the question of whether such strong electron-phonon coupling is realized in cuprates. Strong coupling with some collective excitation mode has been indicated by a dispersion “kink”. However, there is intensive debate regarding whether the relevant coupling mode is a magnetic resonance mode or an oxygen buckling phonon mode. This ambiguity is a consequence of the energy of the main prominent kink. Here, we show a new landscape of dispersion kinks. We report that heavily overdoping a Bi_2_Sr_2_CaCu_2_O_8+*δ*_ superconductor results in a decline of the conventional main kink and a rise of another sharp kink, along with substantial energy shifts of both. Notably, the latter kink can be ascribed only to an oxygen-breathing phonon. Hence, the multiple phonon branches provide a consistent account of our data set on the multiple kinks. Our results suggest that strong electron-phonon coupling and its dramatic change should be incorporated into or reconciled with scenarios for the evolution of high-*T*_c_ superconductivity.

## Introduction

To identify the pairing mechanism of superconductivity, it is crucial to inspect the fingerprints of the collective excitation modes coupled with an electron. Indeed, a phonon-mediated scenario for conventional superconductivity was established by showing that the modulation pattern of tunneling spectra is consistent with the energy distribution of the electron-phonon coupling^1, 2^. Angle-resolved photoemission spectroscopy (ARPES) has served as a momentum-resolved probe of the coupling strength distribution, and it has been reported that the electronic dispersion of high-*T*_c_ cuprates usually has a prominent “kink” at around *ω* ~ 65 meV^3–6^. This has been ascribed to strong coupling with the bond-buckling B_1*g*_ phonon of CuO_2_ planes^7–11^ or the magnetic resonance mode detected by inelastic neutron scattering (INS)^12–14^. Recent ARPES studies of Bi_2_Sr_2_CaCu_2_O_8+*δ*_ (Bi2212) reported that the energy of the prominent kink is largely anisotropic^15, 16^. In going from an antinodal to a nodal region, the prominent kink shifts continuously in energy by ~30 meV for overdoped Bi2212^15^. However, the origin of this strongly anisotropic kink energy is unclear in terms of the phonon and magnon excitations. Further, the detection of additional kinks has proven that the scenarios should be refined for a comprehensive understanding^15, 17, 18^. Therefore, an extensive dependence study with full resolution of the multiple kinks is needed to clarify the factors involved in the high-*T*_c_ superconductivity.

Heavily overdoped Bi2212 provides us with a unique opportunity. First, one can drastically reduce the magnitude of the superconducting gap without increasing the temperature^19, 20^. This is ideal for studying the origin of the dispersion kink, because the kink energy depends not only on the boson frequencies but also on the electronic excitation^8, 9^. Such data provide a clue to the momenta of the electronic states involved. Second, the width of spectral peak decreases with overdoping in the cuprates. This is helpful in resolving the multiple kinks in dispersion. Third, being away from the antiferromagnetic phase boundary simplifies the analysis. Specifically, INS in overdoped La_2−*x*_Sr_*x*_CuO_4_ has shown suppression of the broad magnetic excitations around 40–70 meV, namely, the energies relevant to the prominent kink^21, 22^. These peculiarities are noted in light of the fine resolution of low-energy ARPES.

In this Letter, we report a comprehensive kink study of heavily overdoped Bi2212 using low-energy synchrotron-radiation ARPES. We show that such overdoping noticeably affects the landscape of the kinks. Specifically, we find two prominent kinks well-separated in energy and demonstrate their rise, decline, and energy shifts with overdoping. In view of the distribution of phononic and magnetic excitations, the rising kink is interpreted only as electron coupling with the in-plane bond-stretching breathing mode. We argue that a strong electron-phonon coupling is needed to explain the results.

## Results

**Figure 1:**
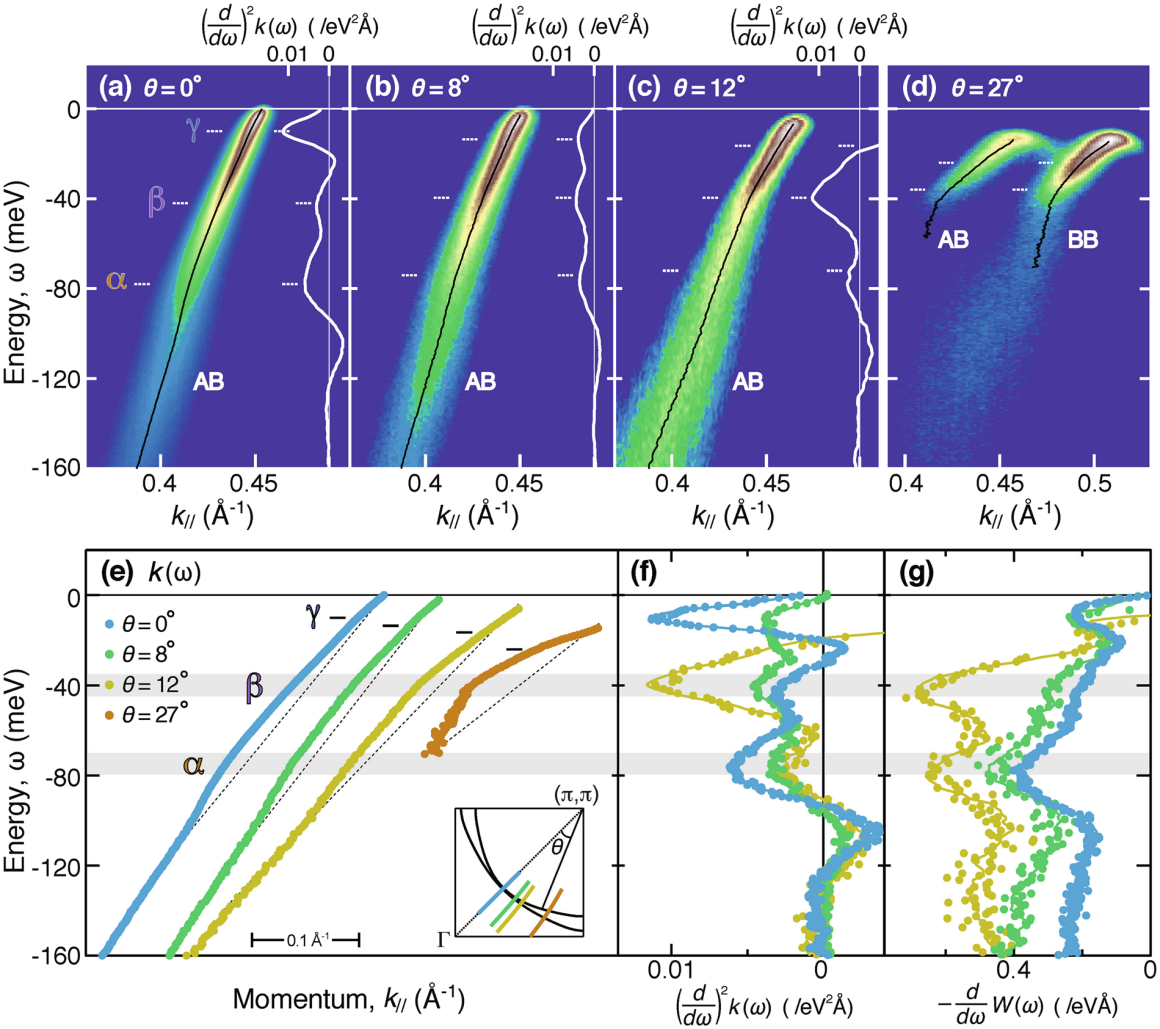
Low-energy ARPES data of heavily overdoped Bi2212 in superconducting state at 10 K. (**a**–**d**) Energy-momentum plots taken at off-node angles of *θ* = 0°, 8°, 12°, and 27°, as marked in the inset of panel (**e**). Black and white curves denote the MDC peak dispersion, *k*(*ω*) (bottom axis), and its second energy derivative, (*d*/*dω*)^2^ *k*(*ω*) (top axis), respectively. In panel (**d**), the bonding band (BB) and antibonding band (AB) are simultaneously observed with *hν* = 8.5 eV, whereas in panels (**a**–**c**), the AB is selectively observed with *hν* = 7.0 eV^19^. (**e**) Offset plot of the dispersion, *k*(*ω*), taken from (**a**–**d**). Dashed lines are guides to the eye. Three kinks are labelled *α*, *β*, and *γ* from high to low binding energies. (**f**) Superimposed plot of (*d*/*dω*)^2^ *k*(*ω*) taken from (**a**–**c**). (**g**) Energy derivative of MDC peak width, −*dW*(*ω*)/*dω*.

Figure 1(a–d) show the ARPES spectra along representative cuts for a heavily overdoped Bi2212 sample with *T*_c_ = 63 K (OD63). In this study, the bilayer bands are resolved throughout the Brillouin zone by using *hν* = 8.5 eV, and the antibonding band is selectively observed by using *hν* = 7.0 eV^18–20^. This allows us to minimize the uncertainties arising in the data analysis of the spectra of the bilayer-split bands. Black curves in Fig. 1(a–d) denote the quasiparticle dispersion, *k*(*ω*), determined from fitting analysis of the momentum distribution curve (MDC). Figure 1(a) shows the nodal cut, which reveals that the group velocity, *dω*/*dk*, changes abruptly at energies of |*ω*| = 78, 42, and 10 meV, as clearly seen from the peaks in the second energy derivatives, (*d*/*dω*)^2^ *k*(*ω*) (white curves). The differential coefficient was evaluated in an energy window by using a least-squares linear regression method (solid curves) and by taking the simple difference between the values at both ends of the window (filled circles)^18, 20^ (see methods section). Hereafter, we call these kinks *α*, *β*, and *γ* from high to low binding energies. Note that both the *α* and *β* kinks differ significantly in energy from the well-known prominent kink that has been observed at 65 meV for optimum doping^3, 4, 18^.

**Figure 2:**
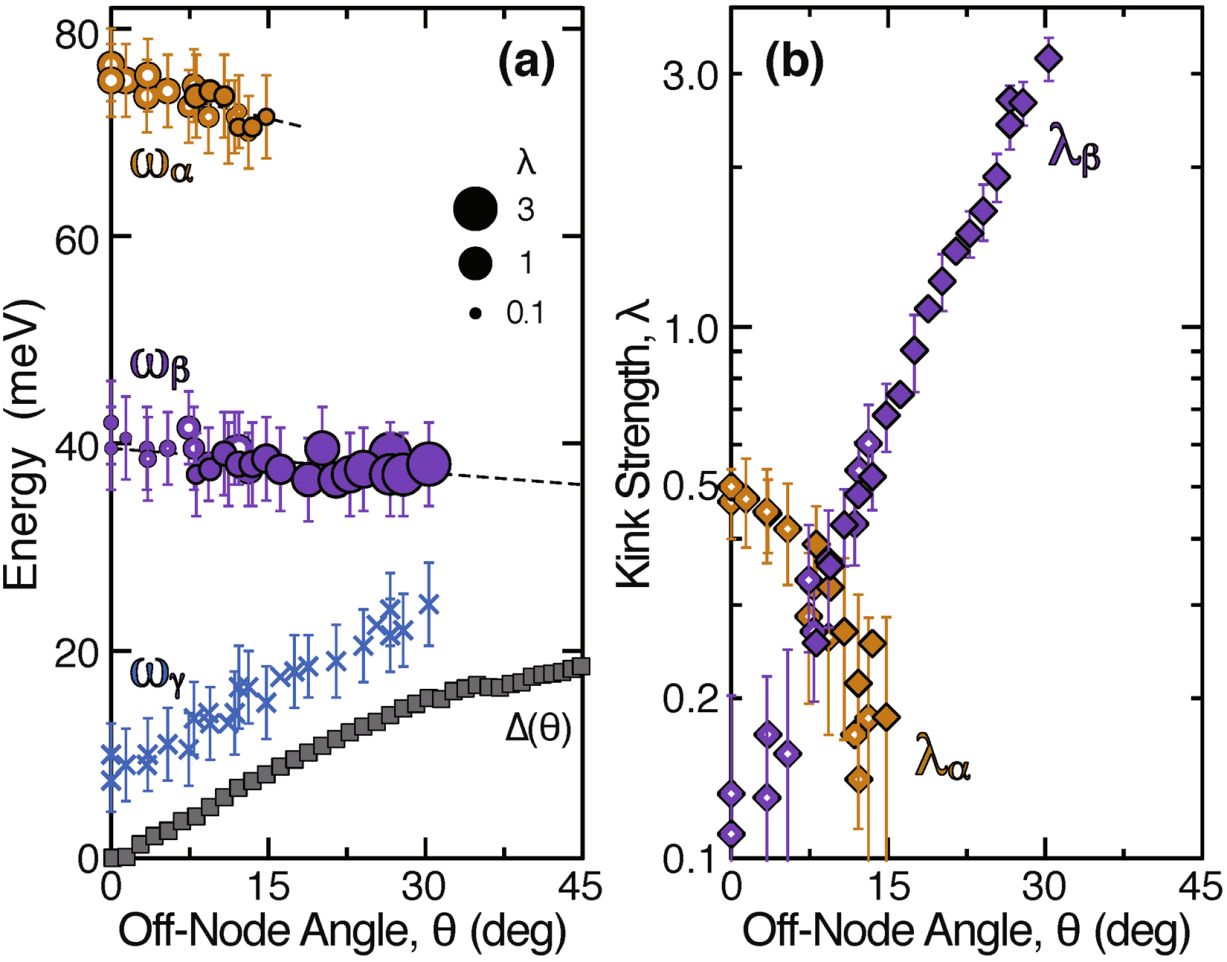
Dispersion kinks as functions of *θ*, observed at 10 K for OD63. Open and filled symbols represent data taken with *hν* = 7.0 and 8.5 eV, respectively. (**a**) Kink energies *ω*_*α*_ (brown circles), *ω*_*β*_ (purple circles), and *ω*_*γ*_ (blue crosses) determined from (*d*/*dω*)^2^ *k*(*ω*). Circle size represents the kink strength, and dashed lines denote the linear fits. The error bars for *ω*_*α*_, *ω*_*β*_, and *ω*_*γ*_ represent the uncertainty in determining the peak position of (*d*/*dω*)^2^ *k*(*ω*). Also shown are the superconducting gap Δ(*θ*) (grey squares)^19^. (**b**) Kink strengths *λ*_*α*_ = *v*_100_/*v*_60_ − 1 (brown diamonds) and *λ*_*β*_ = *v*_60_/*v*_25_ − 1 (purple diamonds), where *v*_100_, *v*_60_, and *v*_25_ denote the experimental group velocities at 100, 60, and 25 meV, respectively. The error bars for *λ*_*α*_ and *λ*_*β*_ reflect the uncertainty in determining the group velocity on the high- and low-energy sides.

Even away from the node, the three kinks remain resolved, as shown in Fig. 1(b–e). A superimposed plot of (*d*/*dω*)^2^ *k*(*ω*) in Fig. 1(f) reveals that the *α* and *β* kinks shift little in energy as the off-node angle *θ* changes, despite the dramatic weight transfer between them. This finding is consistent with the features in the energy derivative of the MDC peak width, −*dW*(*ω*)/*dω*, as shown in Fig. 1(g). The energy and strength of the kink were determined from the peak position in (*d*/*dω*)^2^ *k*(*ω*) and from the ratio of the group velocities on the high- and low-energy sides as *λ* = *v*_H_/*v*_L_ − 1, respectively, and are presented in Fig. 2(a,b) ^23^. These plots clarify that the *α* and *β* kinks extensively coexist about 34 meV apart, and their maximum strengths, *λ*_*α*_ = 0.5 and *λ*_*β*_ = 3.2, are observed in the nodal and antinodal regions, respectively, indicating a difference in their coupling modes. The energy of the *γ* kink shifts with *θ* in parallel to the gap opening as ${\omega }_{\gamma }(\theta )\simeq {\rm{\Delta }}(\theta )+10$ meV.

**Figure 3:**
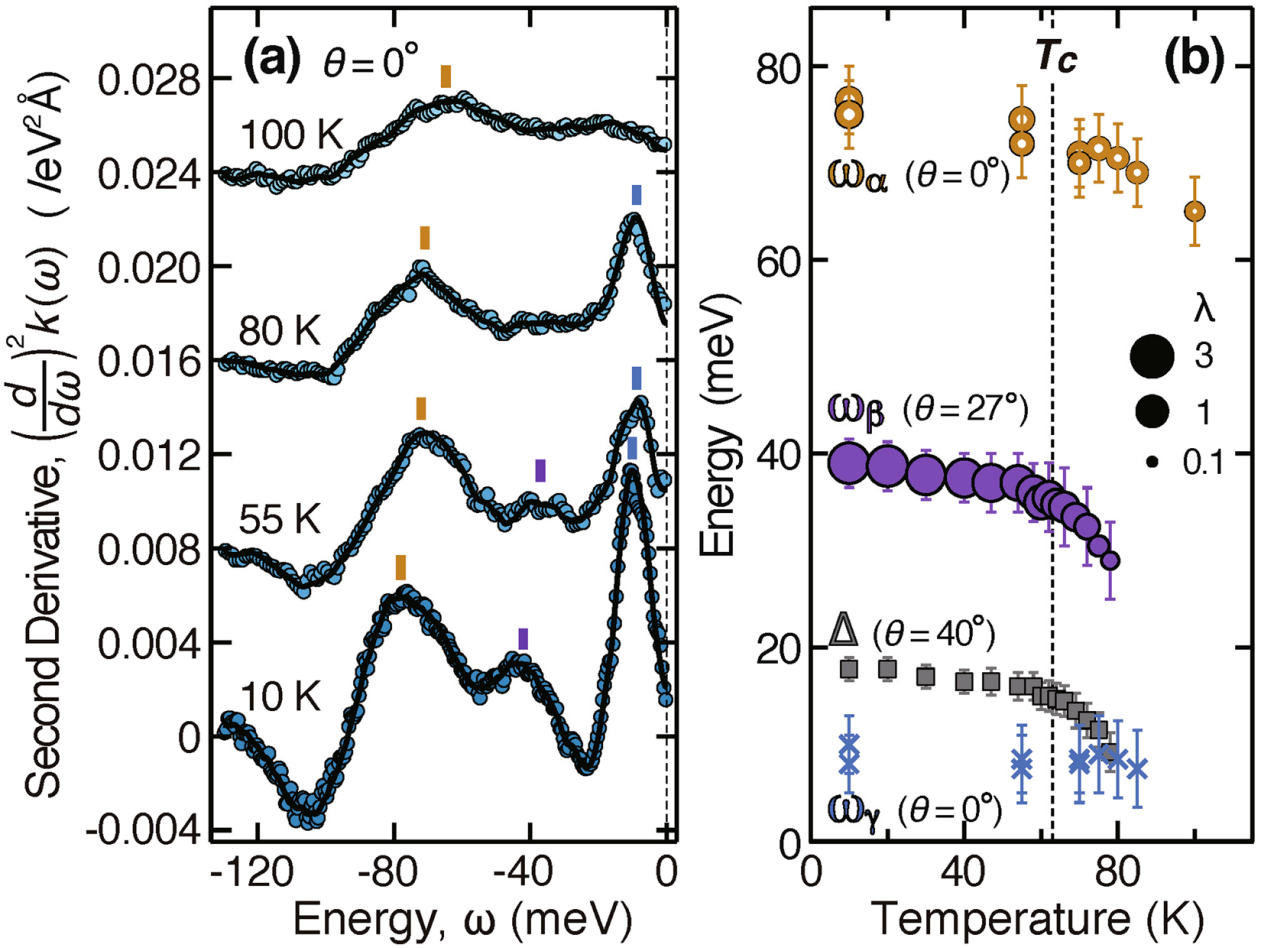
Temperature dependence of OD63. (**a**) Second energy derivative of nodal dispersion, (*d*/*dω*)^2^ *k*(*ω*), at *T* = 10, 55, 80, and 100 K, offset from each other by 0.008 eV^−2^Å^−1^. (**b**) Energies of a nodal *α* kink at *θ* = 0° (brown open circles), an off-nodal *β* kink at *θ* = 27° (purple filled circles), a nodal *γ* kink at *θ* = 0° (blue crosses), and an antinodal gap at *θ* = 40° (grey squares), observed across *T*_c_ = 63 K. Circle size represents the kink strength. The error bars for the kink energy and gap energy represent the uncertainty in determining the peak position of (*d*/*dω*)^2^ *k*(*ω*) and the EDC at the Fermi momentum, respectively.

With increasing temperature *T*, significant shifts in the kinks are observed, as shown by (*d*/*dω*)^2^ *k*(*ω*) in Fig. 3(a). Therefore, we plotted the energies and strengths of the nodal *α* kink (*θ* = 0°) and the off-nodal *β* kink (*θ* = 27°) as functions of *T* in Fig. 3(b). Also shown is the antinodal gap energy, Δ, evaluated at *θ* = 40° from the peak positions of the energy distribution curve (EDC) at the Fermi momentum. A pseudogap appears to remain up to a temperature somewhat higher than *T*_c_. This remaining gap in the overdoped region is consistent with observations in recent high-resolution ARPES studies^24, 25^. Notably, the kink and gap energies, *ω*_*α*_, *ω*_*β*_, and Δ, exhibit similar behavior in Fig. 3(b). All of these energies do not change greatly below *T*_c_ but start to decrease as the temperature exceeds *T*_c_. The parallel curves in Fig. 3(b) indicate that *ω*_*α*_ − Δ and *ω*_*β*_ − Δ are almost constant, suggesting that *ω*_*α*_ and *ω*_*β*_ depend directly on Δ.

**Figure 4:**
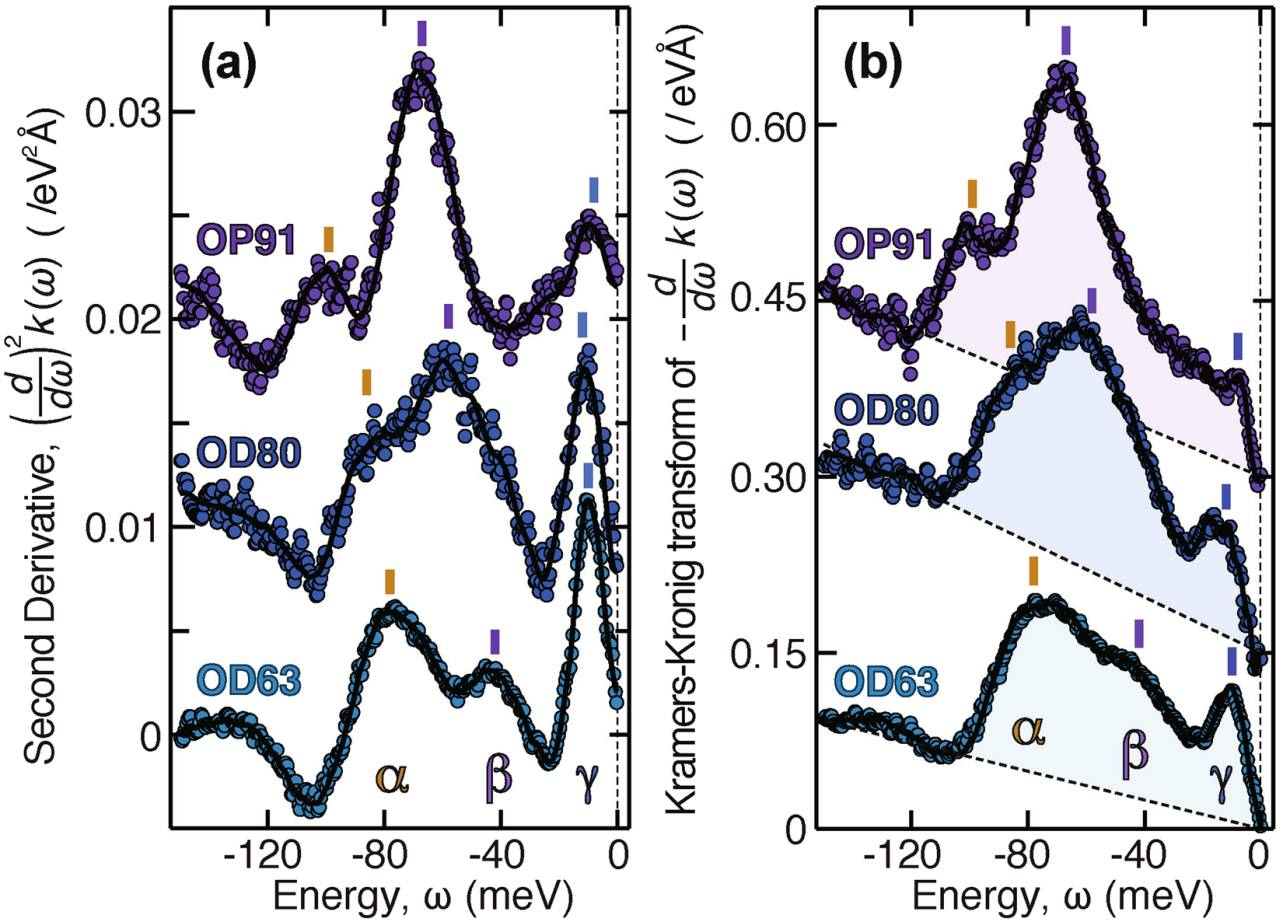
Doping dependence of nodal data. (**a**) Second energy derivatives of dispersion, (*d*/*dω*)^2^ *k*(*ω*), offset from each other by 0.01 eV^−2^Å^−1^. (**b**) Kramers-Kronig transform of inverse group velocity, (*d*/*dω*)*k*(*ω*), offset from each other by 0.15 eV^−1^Å^−1^. The original data taken with *hν* = 8.1 eV for optimally doped *T*_c_ = 91 K (OP91) and overdoped *T*_c_ = 80 K (OD80) samples were reported in ref. 18.

Our findings on OD63 shed new light on previous data. In Fig. 4, the nodal data reported in ref. 18 for optimally doped *T*_c_ = 91 K (OP91) and moderately overdoped *T*_c_ = 80 K (OD80) samples are compared with those for OD63. Here, we focus on the data derived from the dispersion *k*(*ω*) rather than those from the MDC width *W*(*ω*), because the latter are broadened in energy more rapidly than the former by the increase in the spectral width. Owing to the sharp spectral peak of OD63, the multiple structures survive even in −*dW*(*ω*)/*dω* as shown in Fig. 1(g). With moderate doping, however, a tiny feature in −*dW*(*ω*)/*dω* is broadened out as seen from Fig. 2(f) of ref. 18. Figure 4(a,b) show the second derivative of the dispersion, (*d*/*dω*)^2^ *k*(*ω*), as in Figs 1(f) and 3(a), and the Kramers-Kronig transform of the inverse group velocity, (*d*/*dω*)*k*(*ω*), as in ref. 18, respectively. The latter is practically regarded as the coupling strength distribution as a function of the quasiparticle energy *ω* ^18, 20^. At first glance, dominant structures appear at 67 and 58 meV for OP91 and OD80, respectively, which are consistent with the established view^3, 4^. Nevertheless, upon closer inspection of Fig. 4(a,b), one finds a subpeak at 99 meV for OP91 and a shoulder at 86 meV for OD80. It follows that the coexistence of two kinks continues from OP91 to OD63 along with a constant separation of $\simeq 32$ meV.

Now, we examine the possible evolution with doping. In view of the nodal spectra, it may be tempting to associate the *α* kink in OD63 with the well-known main kink at 67 meV in OP91, but this raises problems. First, it is difficult to explain the increase in energy from 67 to 78 meV with overdoping, because the kink energy is given by the sum of the bosonic mode energy Ω and the electronic gap energy at an intermediate-state momentum ***k***_m_ as *ω* = Ω + Δ(***k***_m_). According to the experiments, overdoping leads to an overall reduction by one-half in the superconducting gap energy Δ, whose antinodal values are 38 and 18 meV for OP91 and OD63, respectively^19^, and also to a slight decrease in the energies Ω of the relevant phonon and magnetic resonance modes^26–28^. Second, the subkink in OP91 and the *β* kink in OD63 are out of place in Fig. 4 unless intricate branches of evolution are assumed. Third, the main kink observed at moderate doping levels is strongest around the antinode^7^. This is inconsistent with the momentum dependence of *λ*_*α*_ in Fig. 2(b), but rather consistent with that of *λ*_*β*_. Instead, associating the *β* kink in OD63 with the main kink in OP91, we obtain a clear-cut solution. Specifically, as an effect of the one-half reduction in the gap, the main and subkinks in OP91 are both shifted by 23 meV and evolve into the *β* and *α* kinks in OD63, respectively. On this basis, one can see from Fig. 4 that the *β* and *α* kinks decline and rise, respectively, upon overdoping.

**Figure 5:**
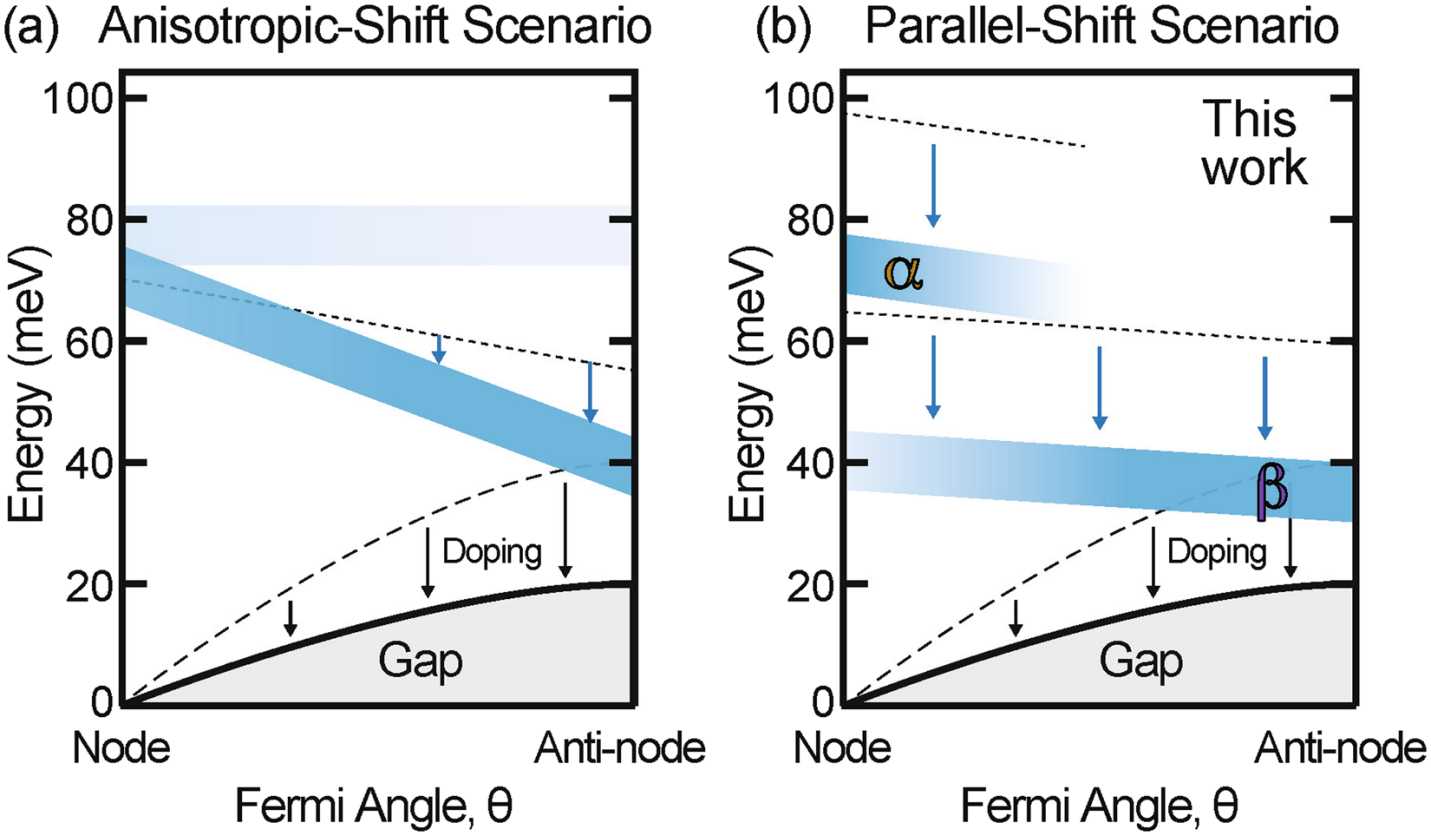
Two pictures of the main kinks in overdoped Bi2212. The strength of the kink is denoted by blue shading. (**a**) Continuous main kink, whose energy shift with overdoping is highly anisotropic, proposed in ref. 15. (**b**) Two separate kinks, whose energy shifts with overdoping are parallel and nearly isotropic, based on the present result.

Two pictures of these kinks are contrasted in Fig. 5. Figure 5(a) shows that a continuous main kink with an anisotropy of ~30 meV was previously reported for an overdoped Bi2212^15^. However, the mechanism by which this anisotropic energy survives against integration over ***k***_m_ has been unclear from the excitation modes^29–31^. In the present study, further overdoping resulted in sharpening of the kinks (Fig. 4) and revealed that the two kink energies coexist in a broad nodal region for OD63, as typified by the nodal kinks at ${\omega }_{\alpha }\simeq 78$ meV and ${\omega }_{\beta }\simeq 42$ meV. Hence, we found that they are less anisotropic than the kink reported by the previous studies^15, 16^. Figure 5(b) shows that our revised picture is simply described by parallel and isotropic shifts of *ω*_*α*_ and *ω*_*β*_ with overdoping.

## Discussion

The observed effects of temperature and overdoping in the gap energy are consistent. As seen from Figs 3 and 4, the decreases in *ω*_*α*_ and *ω*_*β*_ keep pace with that in Δ, whether the gap reduction is caused by temperature or overdoping. These data provide experimental evidence that the antinodal states play a major role in the intermediate electronic channels leading to the *α* and *β* kinks. We note that this interpretation is independent of a pseudogap or superconducting gap. The antinodal states are typically expected to have a key role owing to the *d*-wave-like gap anisotropy, ${\rm{\Delta }}(\theta )\propto \,\sin \,\theta$. Given a certain window for the intermediate momentum ***k***_m_, the process through the nodal states is broadened in energy with the steepest gap slope, whereas that through the antinodal states is hardly affected without the gap slope and thus dominates the peak in (*d*/*dω*)^2^ *k*(*ω*)^8, 20^. Considering that the gap is less than 20 meV for OD63^19^, and that the kink shifts of 10 meV are indeed observed with increasing temperature above *T*_c_, we deduce that there should be two coupling modes whose energies are 10–20 meV lower than *ω*_*α*_ and *ω*_*β*_ and separated by $\simeq 32$ meV.

In light of this, we survey the magnetic excitation. The fundamental energy is given by the (*π*, *π*) resonance mode, from which steep upward and downward dispersions extend in an hourglass shape^28^. The resonance peak is at 34 meV for overdoped Bi2212^32^ and thus can be assigned to the *β* kink. However, the steep part of the dispersion has no energy assignable to the *α* kink^32, 33^; furthermore, the INS spectral weight of magnetic excitation disappears from 40 < *ω* < 70 meV with heavy overdoping^21, 22^. Therefore, something else is required to explain the *α* kink.

Regarding the phonon excitations, the importance of the two vibration modes of the CuO_2_ planes has been argued^3, 9, 10^. The in-plane bond-stretching breathing mode has to do with electrons, as manifested by doping-induced softening^3, 27, 29^. The out-of-plane bond-buckling B_1g_ mode is predicted to make the largest contribution to electron-phonon coupling^9, 34^. The former and latter modes have been detected at about 65 and 35 meV, respectively, for overdoped Bi2212 by Raman scattering experiments^26, 35^, and are expected to be coupled mainly to the nodal electrons by *λ* ~ 0.3 and to the antinodal electrons by *λ* ~ 2.5, respectively^9^. Therefore, the breathing and buckling phonons correspond to the nodal *α* and antinodal *β* kinks, respectively, and give a comprehensive account of our data for *ω*_*α*_, *ω*_*β*_, *λ*_*α*_(*θ*) and *λ*_*β*_(*θ*) in Fig. 2.

Next, we further discuss the reality of the phonon-based kink model. Because of the difficulty in assigning the *α* kink to the magnetic excitations, our observation of *α* kink provides a compelling evidence that the electron-phonon coupling is indeed strong enough to cause the appreciable dispersion kink in the cuprates. On the other hand, it has been reported that the electron-phonon coupling in the density-functional-theory (DFT) calculation is too weak to reproduce the experimental dispersion kink^10, 36^. However, it has also been argued that the DFT calculation tends to overestimate screening effect and thus to underestimate the electron-phonon coupling^11, 34^. In the experiments so far, an early ARPES study aroused keen interests in isotope effect on the dispersion kink^37^, because the isotope atomic mass makes energy shifts of the phonon modes^38^ without affecting the magnetic excitations. Subsequently, a low-energy ARPES study has revealed a reasonable amount of isotope shift of the *β* kink energy *ω*_*β*_ at the node, proving the involvement of phonons^6^. Furthermore, scanning tunneling experiments have shown that the spectral feature corresponding to the *β* kink is shifted in energy by the isotope substitution^39^, as reproduced by a strong-coupling theory^40^. These isotope experiments and the presence of distinct *α* kink consistently indicate that the coupling with phonon makes a major impact on the electronic excitation spectra.

For the origin of the *β* kink, the buckling phonon and magnetic resonance modes possibly overlap with each other, because their energies are similar. Here, the *α* kink arising solely from the breathing phonon mode can serve as a reference of the electron-phonon coupling. In the model calculation cited above from ref. 9, the buckling mode coupling at the antinode is about 8 times larger than the breathing one at the node. As shown in Fig. 2, our experimental data of relative kink strength, *λ*_*β*_/*λ*_*α*_ ~ 6.4, is not more than the phonon-model prediction. Hence, the contribution from the magnetic resonance mode is constrained to a minor part of the presently observed *β* kink, and we infer that the major part is the contribution from the buckling phonon mode.

Concerning the *γ* kink, the functional form of kink energy, ${\omega }_{\gamma }(\theta )\simeq {\rm{\Delta }}(\theta )+10$ meV, has been obtained for heavily overdoped Bi2212. This is qualitatively consistent with the previous reports on underdoped and optimally doped Bi2212, and thus the *γ* kink is ascribed to the effect of forward scattering by low-frequency phonons or out-of-pklier^18, 41, 42^.

In conclusion, we depicted the doping- and temperature-dependent landscape of the dispersion kinks in a cuprate superconductor. In consequence of heavy overdoping, a rising *α* kink and a declining *β* kink are observed at 78 and 42 meV, respectively, in a nodal cut. The distinctive point is that two well-separated prominent kinks coexist in a broad nodal region for heavily overdoped Bi2212. The resultant data provide evidence that these two kink energies are governed by the antinodal gap as well as the coupling mode. It follows that the *β* kink is ascribed to the bond-buckling phonon or the magnetic resonance mode. Nevertheless, the *α* kink is difficult to explain without the bond-stretching breathing phonon, because there is no well-defined magnetic excitation at this energy. The quantitative evidence of strong electron-phonon coupling and its radical doping dependence will play an essential role in accounting for the evolution of electron pairing in the high-*T*_c_ cuprates.

## Methods

### Experimental details

High-quality single crystals of heavily overdoped Bi_1.54_Pb_0.6_Sr_1.88_CaCu_2_O_8+*δ*_ with *T*_c_ = 63 K (OD63) were prepared by a traveling-solvent floating-zone method and a post-annealing procedure. ARPES spectra were collected at BL-9A of the Hiroshima Synchrotron Radiation Center using a Scienta R4000 hemispherical electron analyzer. The instrumental energy and momentum resolutions were 5 meV and 0.004 Å^−1^, respectively. The samples were cleaved *in situ*, and kept under an ultrahigh vacuum (better than 5 × 10^−11^ Torr). We adopted two photon energies, *hν* = 7.0 and 8.5 eV, as low-energy excitation photons^18^. By using photons with *hν* = 7.0 eV, one can selectively observe an antibonding band despite small splitting due to the CuO_2_ bilayer, but the spectral intensity drops rapidly with increasing distance from the node^19^. By using *hν* = 8.5 eV, in contrast, one can observe sufficient intensity even in the far-nodal region, where the bonding and antibonding bands are widely separated^19^. Note that the effect of transition-matrix elements between the initial and final photoexcitation states can be ruled out in this study, because the kink energies exhibit similar behavior with respect to the momentum and doping at different photon energies, as shown in Figs 1, 2 and 4.

### Fitting analysis

The quasiparticle dispersion, *k*(*ω*), was determined by fitting the MDCs with the Voigt function including a linear background as a nondispersive component. The Gaussian width was fixed to represent the instrumental resolution of Δ*k* = 0.004 Å^−1^. In this study, we determined the kink energies from the second energy derivative spectra of *k*(*ω*). The quasiparticle dispersion, *k*(*ω*), is given by *k*(*ω*) = [*ω* − ReΣ(*ω*)]/*v*_0_, where Σ(*ω*) is the real part of the self-energy, and *v*_0_ is the bare band velocity. Using a constant velocity *v*_0_ as the hypothetical bare electron dispersion, the first derivative of the quasiparticle dispersion is written as (*d*/*dω*)*k*(*ω*) = [1 − (*d*/*dω*)ReΣ(*ω*)]/*v*_0_. The peak feature of *k*(*ω*) is described as the step-like increase in the (*d*/*dω*)*k*(*ω*). Taking the second energy derivatives of *k*(*ω*), we obtain the formula (*d*/*dω*)^2^ *k*(*ω*) = −(*d*/*dω*)^2^ReΣ(*ω*)/*v*_0_. Thus, the peaks in ReΣ(*ω*) are determined from the second energy derivative of the quasiparticle dispersion *k*(*ω*). The peak positions of the (*d*/*dω*)^2^ *k*(*ω*) shown in Figs 1, 3 and 4 can be assigned as the energy positions of the kinks.

The differential coefficient at a certain energy point *ω* was evaluated in an energy window from *ω* − *E*_W_(*ω*) to *ω* + *E*_W_(*ω*), by using a least-squares linear regression method (solid curves) and by taking the simple difference between the values at both ends of the window (filled circles) in Figs 1(a–c,f,g), 3(a) and 4(a,b). The window-width function of *E*_W_(*ω*) = *A*|*ω*| + *B* is varied slightly depending on the signal-to-noise ratio of the ARPES spectra from the nodal to the antinodal region, where the coefficients *A* and *B* are in the ranges 0.15 < *A* < 0.40 and 8.5 < *B* < 12.5 meV, respectively. We carefully confirmed that the characteristic feature of (*d*/*dω*)^2^ *k*(*ω*), i.e., the three peak structures at |*ω*| < 120 meV, are robust and independent of the variation of *E*_W_(*ω*).
