# Publisher Correction: A New Landscape of Multiple Dispersion Kinks in a High-*T*_c_ Cuprate Superconductor

**DOI:** 10.1038/s41598-020-69656-x

**Published:** 2020-08-07

**Authors:** H. Anzai, M. Arita, H. Namatame, M. Taniguchi, M. Ishikado, K. Fujita, S. Ishida, S. Uchida, A. Ino

**Affiliations:** 1 Graduate School of Engineering, Osaka Prefecture University, Sakai, 599-8531 Japan; 2 Hiroshima Synchrotron Radiation Center, Hiroshima University, Higashi-Hiroshima, 739-0046 Japan; 3 Graduate School of Science, Hiroshima University, Higashi-Hiroshima, 739-8526 Japan; 4 Research Center for Neutron Science and Technology, Comprehensive Research Organization for Science and Society (CROSS), Tokai, Naka, Ibaraki 319-1106 Japan; 5 Laboratory for Atomic and Solid State Physics, Department of Physics, Cornell University, Ithaca, NY 14853 USA; 6 Advanced Industrial Science and Technology, Tsukuba, Ibaraki 305-8568 Japan; 7 Department of Physics, University of Tokyo, Tokyo, 113-0033 Japan; 8 Present Address: Condensed Matter Physics and Materials Science Department, Brookhaven National Laboratory, Upton, NY 11973 USA

Correction to: *Scientific Reports*https://doi.org/10.1038/s41598-017-04983-0, published online 06 July 2017.

This Article contains errors in Discussion.

“Concerning the γ kink, the functional form of kink energy, ω_γ_(θ) ≃ Δ(θ) + 10 meV, has been obtained for heavily overdoped Bi2212. This is qualitatively consistent with the previous reports on underdoped and optimally doped Bi2212, and thus the γ kink is ascribed to the effect of forward scattering by low-frequency phonons or out-of-pklier^18,41,42^.”

should read:

“Concerning the γ kink, the functional form of kink energy, ω_γ_(θ) ≃ Δ(θ) + 10 meV, has been obtained for heavily overdoped Bi2212. This is qualitatively consistent with the previous reports on underdoped and optimally doped Bi2212, and thus the γ kink is ascribed to the effect of forward scattering by low-frequency phonons or out-of-plane disorder, following the earlier^18,41,42^.”

